# Assessing the efficacy and safety of different nonsteroidal anti-inflammatory drugs in the treatment of osteoarthritis: A systematic review and network meta-analysis based on RCT trials

**DOI:** 10.1371/journal.pone.0320379

**Published:** 2025-05-07

**Authors:** Pan JiaoYi, Sun YongQi, Guo KeChun, Li XingYu, Liu ZeZhong, Duan Jin Shuai, Gong YouJia, Xu Bing, Wang XiaoFeng

**Affiliations:** 1 Wenzhou Hospital of Integrated Traditional Chinese and Western Medicine Affiliated to Zhejiang Chinese Medical University, Zhejiang University of Traditional Chinese Medicine, Wengzhou, Zhejiang, Peoples R China; 2 The Third Clinical College of Zhejiang University of Traditional Chinese Medicine, Hangzhou, Zhejiang, Peoples R China; 3 Xiangtan Chinese Medical Hospital, Xiangtan, Hunan, Peoples R China; University of Alcalá, SPAIN

## Abstract

**Introduction:**

Osteoarthritis (OA) as a degenerative disease, has seen a continuous rise in incidence and prevalence globally since 1990, imposing a significant disease burden. NSAIDs (Nonsteroidal anti-inflammatory drugs) as symptomatic medications for OA treatment, hold an indispensable position in clinical practice.

**Objective:**

To evaluate the efficacy and safety of different NSAIDs in the treatment of OA through Bayesian Network Meta-Analysis (NMA).

**Methods:**

Randomized controlled trials (RCTs) on NSAIDs for OA treatment were retrieved from PubMed, Web of Science, Embase, and the Cochrane Library databases. The search timeframe was from the inception of each database up to June 1, 2024. Outcome indicators for NMA were all conducted using a random-effects model. MetaInsight and Stata 14.0 software were used in R for calculations and plotting of NMA. Measurement data were represented by mean difference (MD), and count data by odds ratio (OR); a 95% confidence interval (CI) was also calculated for each effect size.

**Results:**

This study included 31 studies, involving 68,539 patients with knee osteoarthritis (KOA) and 16 interventions. NMA results showed that compared to the placebo, Tiaprofenic reduced the VAS score (MD =  -0.16, 95% CI: (-0.46 to 0.14), P >  0.05), albeit without significant difference; meanwhile, Diclofenac reduced the total WOMAC score in KOA patients (MD =  -0.41, 95% CI: -1.05 to 0.24, P >  0.05). Compared to the placebo, Etoricoxib was the best medication for improving the WOMAC pain subscale score (MD =  -0.44; 95% CI: -0.61 to -0.26); Naproxen significantly improved the WOMAC Function score in KOA patients after administration (MD = -0.43; 95% CI: -0.82 to -0.04); Diclofenac intervention significantly reduced the WOMAC Stiffness score in KOA patients (MD =  -0.40; 95% CI: -0.67 to -0.13). In terms of adverse event rates, compared to the placebo, the use of Etoricoxib significantly increased the incidence of cardiovascular adverse events (OR =  0.56, 95% CI: 0.32–0.99); Ketoprofen had fewer gastrointestinal adverse events during the medication process (OR =  0.09, 95% CI: 0.04–0.20); Licofelone had a lower rate of other adverse events during the medication process (OR =  0.80, 95% CI: 0.45–1.40, P >  0.05). Therefore, the results indicate that Etoricoxib, Tiaprofenic, Naproxen, Diclofenac, and Ketoprofen have better clinical efficacy and safety.

**Conclusion:**

Compared to other NSAIDs, Etoricoxib, Tiaprofenic, Naproxen, and Diclofenac play a more effective role in improving clinical symptoms of OA; in terms of reducing the incidence of adverse events, Ketoprofen has a lower chance of adverse events. However, the possibility of these results still needs further clinical and basic research for verification.

## Introduction

Osteoarthritis (OA) is a chronic degenerative joint disease characterized by the degeneration of articular cartilage, synovial inflammation, and subchondral bone lesions [1]. Its pathological features include the infiltration of mononuclear cells into the synovial joints, thereby promoting inflammation, stiffness, joint swelling, cartilage degeneration, and further bone destruction [2]. These pathological changes lead to limited knee joint activity, stiffness, functional impairment, and adversely affect the quality of life of patients [3]. With the aging of the global population and the youthfulness of the disease, 240 million people suffer from symptomatic activity-limited OA, and the incidence is continuously rising. This not only widely affects the quality of life of the elderly but also impacts the work of many young and middle-aged people, greatly increasing the economic pressure on the healthcare system. Currently, drugs used for OA treatment include NSAIDs (Nonsteroidal anti-inflammatory drugs) [4], cartilage-protecting drugs (CP), opioid drugs, and glucocorticoids. Cartilage-protecting drugs cannot inhibit the progression of inflammation; opioid drugs only play an analgesic role in the acute phase and can lead to worse baseline knee joint structure degradation and faster progression [4]; long-term use of glucocorticoids can lead to bone fragility and increase the risk of fractures. The inflammation, pain, and fever manifested by OA are related to the production of pro-inflammatory chemicals known as prostaglandins. NSAIDs reduce the production of prostaglandins by inhibiting cyclooxygenase (COX), thereby playing an anti-inflammatory, analgesic, and antipyretic role [5], making NSAIDs the main choice in the treatment of OA.

Currently, there are many varieties of NSAIDs, commonly including Acetaminophen, Celecoxib, Diclofenac, Etoricoxib, etc. Among them, Acetaminophen is the most commonly used analgesic and is recommended by the World Health Organization (WHO) as the first-line treatment for pain [6], but incorrect use may lead to liver failure. Celecoxib can also treat various types of arthritic pain [7] and has a lower risk of bleeding than other NSAIDs. Diclofenac is a derivative of benzoic acid; it can reduce inflammation, thereby reducing nociceptive pain, but it inhibits the production of protective mucus in the stomach, increasing the risk of gastrointestinal ulcers [8]; Etoricoxib is a selective COX-2 inhibitor (with a selectivity for COX-2 inhibition about 106 times that of COX-1), compared with the placebo, treatment with COXIB can increase the incidence of cardiovascular adverse events. Different NSAIDs have different advantages and disadvantages, and how to choose drugs while balancing efficacy and safety has always been a controversial topic.

Despite the wide variety of NSAIDs available, direct comparative studies evaluating their relative efficacy and safety are limited. Most existing studies have focused on comparing one or two NSAIDs, or comparing NSAIDs with other drug classes [9]. For example, Kongtharvonskul [10] conducted a systematic review and network meta - analysis of RCTs to compare the efficacy of NSAIDs in knee osteoarthritis. Bannuru [11] focused on evaluating the relative efficacy of IAHA compared with NSAIDs for knee OA. However, these studies have their own limitations. Specifically, the work of Kongtharvonskul. only covered a limited range of NSAIDs, and Bannuru did not provide a comprehensive comparison in terms of both efficacy and safety.

Our study differs by including more NSAIDs and using Bayesian network meta - analysis, offering a more complete and systematic comparison to fill the knowledge gap. Despite the variety of NSAIDs, direct comparative studies on their relative efficacy and safety are scarce, mostly focusing on one or two NSAIDs or comparisons with other drugs. This hinders clinicians’ evidence - based decision - making in selecting the optimal NSAID for OA treatment. Our study, through Bayesian network meta - analysis, evaluates the clinical efficacy and safety of various NSAIDs, bridging the knowledge gap and supporting rational, scientific drug use in clinical practice.

## Materials and methods

The implementation of this NMA follows the latest 2020 statement of the Preferred Reporting Items for Systematic Reviews and Meta-Analyses (PRISMA) guidelines for systematic review reporting. This study does not require ethical review because all data come from publicly accessible sources. The NMA protocol has been registered in the International Prospective Register of Systematic Reviews (PROSPERO) (registration number CRD42024552748).

### 2.1. Data collection

#### 2.1.1. Inclusion criteria for literature.

All included literature in this study must meet the following requirements: (1) Type of experiment: RCTs (Randomized controlled trials). (2) Patients included in the study must have a confirmed diagnosis of osteoarthritis (OA). The diagnostic criteria used should be clearly stated in the literature, such as those from the American College of Rheumatology (ACR), European League Against Rheumatism (EULAR), and Osteoarthritis Research Society International (OARSI) guidelines. Other criteria that may be used in the evaluated RCTs include clinical symptoms (e.g., joint pain, stiffness) and radiographic findings (e.g., joint space narrowing, osteophytes). (3) Intervention measures: Patients in the experimental group must take oral NSAIDs without other drug treatments. In addition, this study also excludes efficacy studies of combined treatments including exercise or physical therapy; this limited scope will help more accurately assess the efficacy and safety of NSAIDs in the treatment of OA. (4) Control group patients can take oral placebo, hyaluronic acid, chondroitin, etc. In addition, this study allows the inclusion of RCTs for multiple group comparisons. (5) Outcome indicators: Western Ontario and McMaster Universities Osteoarthritis Index (WOMAC) score, visual analog scale (VAS) pain score, adverse events (AE). Literature must report at least one of the above outcome indicators to be included in this study. In addition, the measurement data of continuous variables need to be presented in the form of mean ±  standard deviation, otherwise they are considered not to meet the inclusion criteria. This requirement helps to improve the statistical efficiency of NMA. (6) The language of the literature is not restricted.

#### 2.1.2. Exclusion criteria for literature.

(1) Lack of original data, unextractable, incomplete or incorrect research data (2) Reviews and conferences (3) Repeated publication of the same population’s research data (4) Animal experiments or self-control experiments.

### 2.2. Literature search strategy

As of June 1, 2024, we systematically searched PubMed, Web of Science, Embase, and the Cochrane Library databases using a computer to obtain all publicly available literature related to the research topic of this study since the establishment of each database. To ensure a high recall rate, the search terms were composed of a combination of theme words and free words related to OA and NSAIDs. The search terms included (Osteoarthritis or Osteoarthritis, Spine or Osteoarthritis, Knee or Osteoarthritis, Hip or osteoarthritis, OA or OA, osteoarthritis) and (NSAIDs or Nonsteroidal Antiinflammatory Drugs or Knee Osteoarthritis Non steroidal anti-inflammatory drugs or non-steroidal anti-inflammatory drugs or non-steroidal anti-inflammatory drugs, NSAIDs).

### 2.3. Literature screening and data extraction

Endnote X9 software was used for literature management. After removing duplicate literature, two researchers (JY and XY) independently read the titles and abstracts according to the preliminary screening inclusion and exclusion criteria, and then read the full text of the literature that met the conditions to determine whether to include it. If there were different opinions, discussions were held or the third researcher (ZZ) made a judgment. For the literature finally determined to be included, data extraction was carried out using Excel 2021 software, including (1) general information: literature title, first author, author’s country, publication time, etc.; (2) baseline characteristics: sample size, gender, age, etc.; (3) intervention measures: drug name, control group drug, etc.; (4) bias risk assessment elements; (5) outcome indicators and measurement data.

### 2.4. Bias risk assessment of included literature

We used the Cochrane Risk of Bias Tool 2.0 (ROB2) provided by the Cochrane Systematic Reviewers’ Handbook (6.4 2023 version) to assess the methodological quality and bias risk of the included RCTs. The assessment mainly includes the risk of bias generated in the randomization process, the risk of bias caused by deviations from the expected intervention measures, the risk of bias caused by missing outcome data, the risk of bias in the measurement of results, and the risk of bias in the selection of reported results. Each included RCT can be judged as low risk, high risk, or unclear.

### 2.5. Statistical analysis method

Stata 17.0 was used for Bayesian network meta-analysis. Binary variables were represented by the odds ratio (Odds ratio, OR) as the effect analysis statistic, and continuous variables were represented by the mean difference (Mean difference, MD), and a 95% confidence interval (Confidence interval, CI) was calculated. If the units of continuous variables are not unified, the standardized mean difference (Standardized mean difference, SMD) is used to eliminate differences. When drawing the network evidence map, the size of each point represents the trial scale of the corresponding intervention method, and the strength of the connection between different intervention methods reflects the number of RCTs comparing the two intervention methods. If the network map has an open loop structure, the consistency model is selected. If the network map has a closed loop structure, the consistency of the outcome indicators is tested by the inconsistency test. When P >  0.05, it indicates that there is good consistency between direct evidence and indirect evidence, and the consistency model is used. Subsequently, the surface under the cumulative ranking curve (SUCRA) is calculated to rank different intervention measures. The higher the SUCRA value, the higher the ranking of the drug intervention. According to the SUCRA, the cumulative probability ranking map is drawn to determine the best treatment plan. Comparison-correction funnel plots were used to detect publication bias as well as small sample size effects. When mean and standard deviation values were only available in graphical form, we used WebPlotDigitizer to extract these values. If the data could not be reliably extracted, we attempted to contact the study authors for clarification. If no response was received, the study was excluded from the analysis.

### 2.6. Reporting guidelines and quality assessment

To ensure the rigor and transparency of our meta-analysis, we followed the Preferred Reporting Items for Systematic Reviews and Meta-Analyses (PRISMA) 2020 checklist and the Assessment of Multiple Systematic Reviews (AMSTAR-II) tool. The PRISMA 2020 checklist guided the reporting of our systematic review and meta-analysis, ensuring that all relevant items were addressed in our study design and manuscript preparation. Additionally, we used the AMSTAR-II tool to assess and enhance the methodological quality of our review, particularly in areas such as study selection, data extraction, and risk of bias assessment. A completed PRISMA checklist is available as Supplementary Material (S8 File), and the AMSTAR-II assessment informed the detailed description of our methods and results.

## Results

### 3.1. Results of literature search and basic characteristics

**Fig 1:**
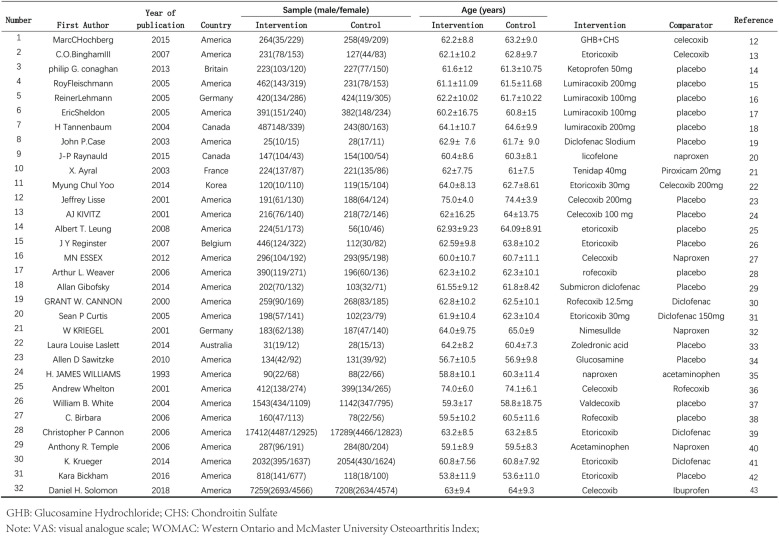
General information on the 32 included studies [12–43].

**Fig 2:**
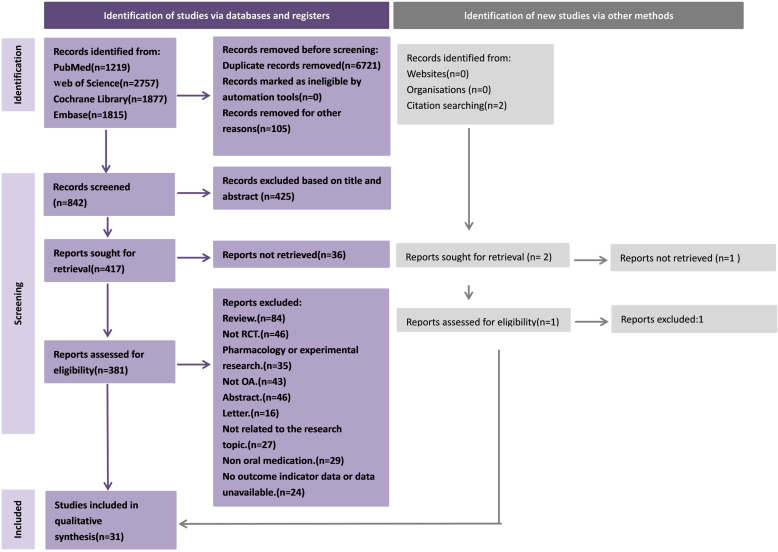
Flowchart of the search and screening process for eligible RCTs.

After the initial search and removal of duplicate articles, we initially obtained 314 articles. After screening according to the inclusion and exclusion criteria, a total of 32 RCTs met the inclusion criteria (Fig 1). The search process and detailed information are shown in Fig 2.

### 3.2. Quality assessment of literature

**Fig 3:**
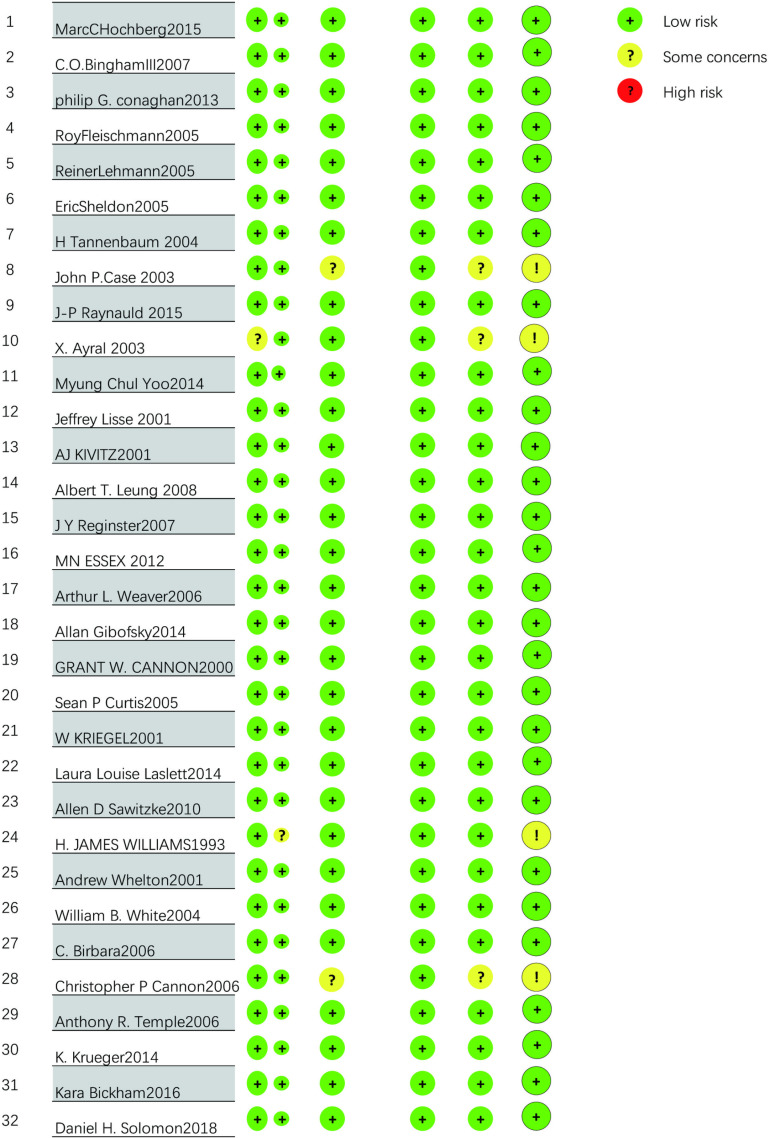
Percentage distribution of risk of bias in randomised controlled trials.

The methodological quality of the 31 RCTs is acceptable, with the vast majority of items assessed as low risk. All 31 used random allocation methods. In the overall evaluation, a total of 2 RCTs] were at high risk. One RCT did not specify specific measures for the randomization process, one RCT did not mention specific deviations from the expected intervention measures, 2 RCTs may have a risk of missing result data, and 4 RCTs were not standardized enough in the selection of reported results, thus being judged as unclear risk. Apart from the above situations, all RCTs were assessed as low risk. The results of the literature quality assessment of the 32 RCTs are shown in Fig 3.

### 3.3. Results of NMA

#### 3.3.1. Consistency test and evidence network diagram.

**Fig 4:**
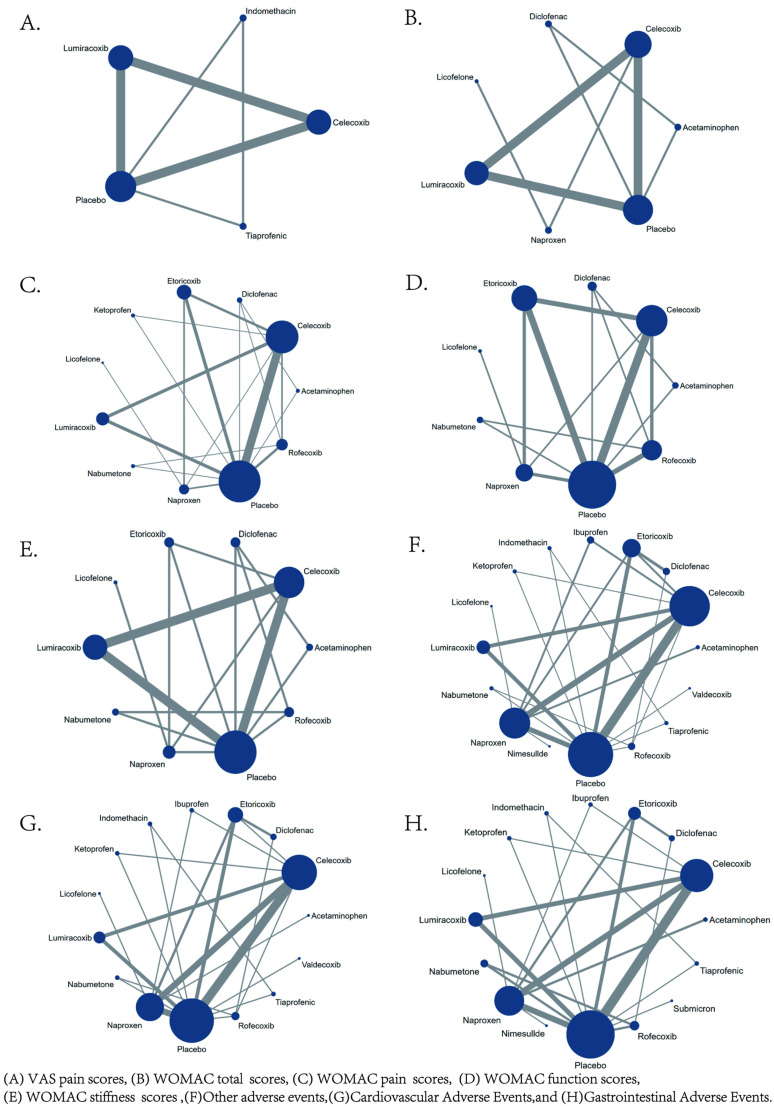
Network evidence.

The network diagrams for the five efficacy outcome indicators and the four safety outcome indicators in this study are all closed-loop structures. The results of the inconsistency analysis show that P >  0.05, indicating no inconsistency in the study, and a consistency model was used for analysis. The network evidence diagrams for VAS pain score, WOMAC total score, WOMAC subscale scores, and AE are shown in Fig 4. In the network evidence diagram, the size of the circle represents the number of patients receiving the intervention, and the thickness of the line between interventions reflects the number of RCTs comparing the two interventions.

#### 3.3.2 Efficacy.

**Fig 5:**
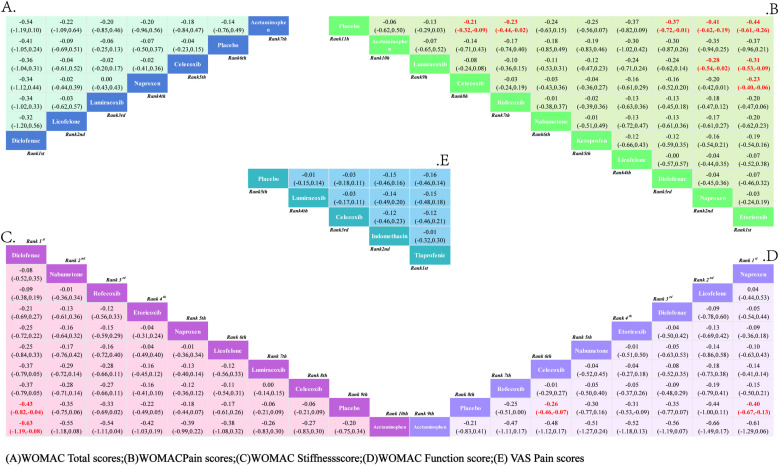
Network meta-analysis.

**3.3.2.1*. VAS pain score*:** A total of 5 RCTs reported comparisons of VAS pain scores, involving 5 interventional medications. The NMA results showed that there were no statistically significant differences in pairwise comparisons of various intervention measures. However, compared to the placebo, Tiaprofenic can reduce the VAS pain score in KOA patients; at the same time, compared with other intervention measures, Tiaprofenic can further alleviate the VAS pain score in KOA patients. The NMA results are shown in Supplementary Material 1 (S1 File). The SUCRA results analysis (Fig 5E) found that compared with the other four interventional medications, Tiaprofenic reduced the VAS pain score in KOA patients to a greater extent.

The SUCRA probability ranking results are Tiaprofenic (72.60%)>  Indomethacin (71.60%)>  Celecoxib (45.50%)>  Lumiracoxib (32.80%)>  Placebo (27.50%). See Appendix.

**3.3.2.2. *WOMAC total score*:** Similarly, a total of 7 RCTs were included, reporting comparisons of WOMAC total scores after administering 7 interventional medications. The NMA results showed that there were no statistically significant differences in pairwise comparisons among the intervention groups. The NMA results are shown in Supplementary material 2(A) (S2 File). The SUCRA results indicate (Fig 5A) that although the difference is not obvious, Diclofenac is the most effective intervention for reducing the total WOMAC score in KOA patients. The SUCRA probability ranking results are: Diclofenac (80.60%)>  Licofelone (48.30%)>  Lumiracoxib (46.90%)>  Naproxen (45.50%)>  Celecoxib (41.90%)>  Placebo (31.60%)>  Acetaminophen (23.10%).

**3.3.2.3. *WOMAC pain score*:** A total of 11 RCTs reported comparisons of WOMAC pain scores, involving 11 interventional medications. The NMA results showed that compared with the placebo, the WOMAC pain score in KOA patients significantly decreased after interventions with Etoricoxib, Naproxen, Diclofenac, Rofecoxib, and Celecoxib, with the most pronounced decrease after Etoricoxib. Secondly, compared with the other 9 interventions, Etoricoxib significantly reduced the WOMAC pain score in KOA patients more than Lumiracoxib and Celecoxib, and although there was no statistical difference compared to the other 7 interventions, the WOMAC pain score was lower after Etoricoxib intervention. The NMA results are in Supplementary material 2(B) (S2 File). The SUCRA results show (Fig 5B) that Etoricoxib is the most effective intervention for reducing the WOMAC pain score in KOA patients, with the probability ranking results being Etoricoxib (84.40%)>  Naproxen (79.60%)>  Diclofenac (71.30%)>  Licofelone (68.20%)>  Ketoprofen (53.10%)>  Nabumetone (51.70%)>  Rofecoxib (50.20%)>  Celecoxib (45.60%)>  Acetaminophen (30.90%)>  Lumiracoxib (30.70%)>  Placebo (10.50%).

**3.3.2.4. *WOMAC Function score*:** A total of 9 RCTs were included, involving comparisons of WOMAC Function scores with 9 interventional medications. The NMA results show that compared to the placebo, Naproxen and Celecoxib significantly improved the WOMAC Function score in KOA patients, with Naproxen showing the most significant improvement. However, when comparing the 8 intervention groups pairwise, no statistical differences were found between the interventions, but Naproxen showed a better improvement. The NMA results are in Supplementary material 2(C) (S2 File). The SUCRA results indicate (Fig 5C) that Naproxen is the most effective intervention for reducing the WOMAC Function score in KOA patients. The SUCRA probability ranking results are Naproxen (72.90%)>  Licofelone (71.00%)>  Diclofenac (63.20%)>  Etoricoxib (57.80%)>  Nabumetone (56.10%)>  Celecoxib (48.90%)>  Rofecoxib (48.20%)>  Placebo (12.10%)>  Acetaminophen (8.50%).

**3.3.2.5. *WOMAC Stiffness score*:** A total of 9 RCTs were included, reporting comparisons of WOMAC Stiffness scores after administering 9 interventional medications. The NMA results show that compared to the placebo intervention, Diclofenac significantly reduced the WOMAC Stiffness score in KOA patients, while other drugs showed no significant statistical differences. When comparing the various drugs statistically, Diclofenac improved the WOMAC Stiffness score significantly better than Acetaminophen and showed no statistical difference compared to the other 7 drugs, but the reduction in WOMAC Stiffness score after Diclofenac was higher than the other 7 drugs. The NMA results are in Supplementary material 2(D) (S2 File). The SUCRA results indicate (Fig 5D) that Diclofenac is the most effective intervention for reducing the WOMAC Stiffness score in KOA patients, with the ranking results being Diclofenac (86.10%)>  Nabumetone (74.60%)>  Rofecoxib (73.90%)>  Etoricoxib (62.50%)>  Naproxen (55.90%)>  Licofelone (52.50%)>  Celecoxib (32.20%)>  Placebo (18.20%)>  Acetaminophen (11.60%).

#### 3.3.3. Safety.

**Fig 6:**
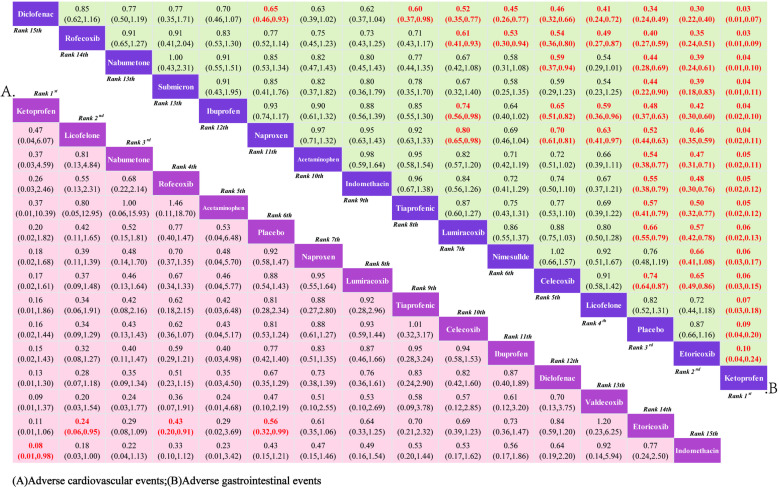
Network meta-analysis.

**3.3.3.1. *Cardiovascular adverse events*:** 15 RCTs reported comparisons of the incidence of cardiovascular adverse events, involving 15 interventional drugs. The NMA results show that compared to the placebo, the incidence of cardiovascular adverse events significantly increased during the use of Etoricoxib. When comparing between groups, the probability of cardiovascular adverse events occurring after Etoricoxib was significantly higher than after Licofelone and Rofecoxib, and although the difference was not statistically significant, Ketoprofen had the lowest incidence of cardiovascular adverse events. The NMA results are in Supplementary material 3 (S3 File). The SUCRA results indicate (Fig 6A) that in the treatment of KOA with drugs, Ketoprofen may be less likely to have cardiovascular adverse events than other treatments; the SUCRA ranking is Ketoprofen (89.90%)>  Licofelone (82.40%)>  Nabumetone (77.9Continuing from where the translation left off:

(90%)>  Rofecoxib (70.80%)>  Acetaminophen (69.00%)>  Placebo (58.20%)>  Naproxen (51.70%)>  Lumiracoxib (47.40%)>  Tiaprofenic (42.70%)>  Celecoxib (39.90%)>  Ibuprofen (36.90%)>  Diclofenac (29.70%)>  Valdecoxib (22.10%)>  Etoricoxib (17.40%)>  Indomethacin (13.80%).

**3.3.3.2. *Gastrointestinal adverse events*:** 15 RCTs were included, covering 15 interventional drugs and reporting comparisons related to gastrointestinal adverse events. The NMA results showed that compared to the placebo, Ketoprofen resulted in fewer gastrointestinal adverse events. When comparing adverse events between drugs, Licofelone, Celecoxib, Nimesulide, Lumiracoxib, Tiaprofenic, Indomethacin, Acetaminophen, Naproxen, Ibuprofen, Nabumetone, Rofecoxib, and Diclofenac were more likely to cause gastrointestinal events than Ketoprofen. The NMA results can be referenced in Supplementary Material 4 (S4 File). Therefore, according to the SUCRA results (Fig 6B), the use of Ketoprofen for the treatment of KOA can better avoid the occurrence of gastrointestinal adverse events. The SUCRA results are Ketoprofen (100.00%)>  Etoricoxib (91.10%)>  Placebo (85.50%)>  Licofelone (73.80%)>  Celecoxib (69.70%)>  Nimesulide (67.50%)>  Lumiracoxib (57.70%)>  Tiaprofenic (44.30%)>  Indomethacin (40.70%)>  Acetaminophen (38.60%)>  Naproxen (35.00%)>  Ibuprofen (27.60%)>  Nabumetone (23.50%)>  Rofecoxib (14.70%)>  Diclofenac (4.40%).

**Fig 7:**
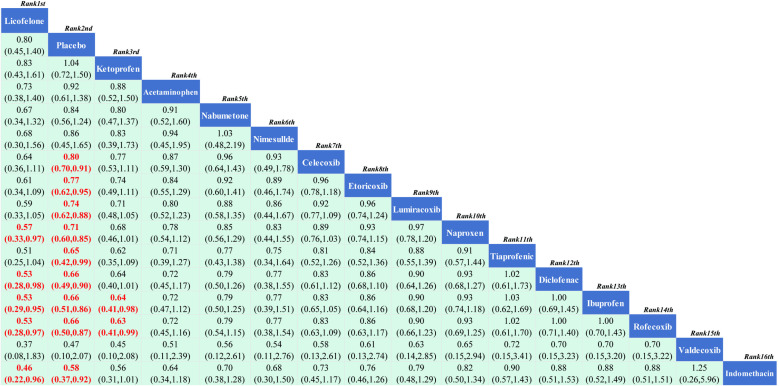
Network meta-analysis.

**3.3.3.3. *Other adverse events*:** A total of 16 RCTs reported comparisons of the incidence of other adverse events, involving 16 interventional drugs. The NMA results showed that compared to the placebo, Licofelone had a lower rate of other adverse events. Compared with the other 14 interventional drugs, Licofelone had a lower probability of other adverse events during treatment. The NMA results are in Supplementary Material 5 (S5 File). The SUCRA results indicate (Fig 7) that during the medication treatment for KOA patients, Licofelone may have a lower incidence of adverse events compared to other treatments; the SUCRA ranking is Licofelone (91.30%)>  Placebo (83.70%)>  Ketoprofen (83.10%)>  Acetaminophen (70.80%)>  Nabumetone (61.70%)>  Nimesulide (59.60%)>  Celecoxib (58.90%)>  Etoricoxib (51.20%)>  Lumiracoxib (43.30%)>  Naproxen (37.00%)>  Tiaprofenic (29.70%)>  Diclofenac (28.10%)>  Ibuprofen (28.00%)>  Rofecoxib (27.60%)>  Valdecoxib (25.80%)>  Indomethacin (20.10%).

#### 3.3.4. Publication bias.

**Fig 8:**
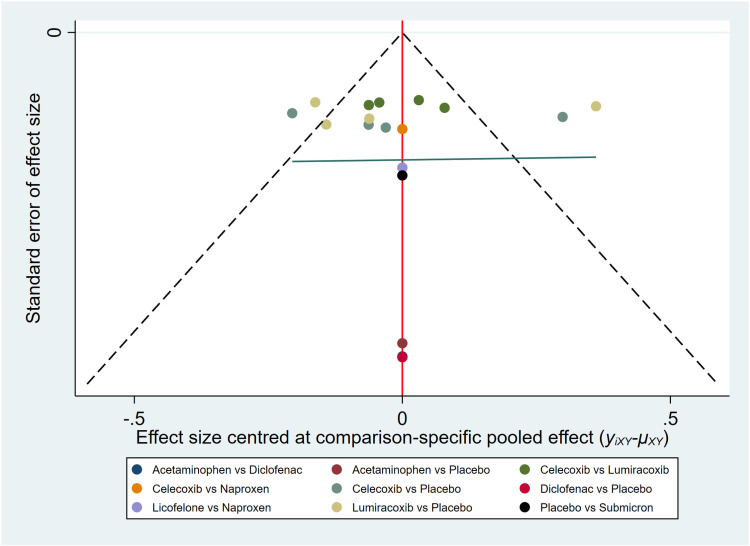
Funnel plot of VAS pain score.

We assessed publication bias using a funnel plot with VAS score as the outcome indicator (Fig 8). After plotting, some points were found to be distributed outside the confidence intervals. The results showed good symmetry for the VAS score outcome indicator, indicating a low likelihood of publication bias.

## Discussion

This is, to our knowledge, the most comprehensive network meta-analysis to assess the efficacy and safety of different nonsteroidal anti-inflammatory drugs (NSAIDs) in the treatment of osteoarthritis (OA), providing an evidence base for clinicians to choose different NSAIDs for the treatment of OA.

From the perspective of pain symptom relief, relevant meta-analysis results have shown that among all topical NSAIDs, diclofenac is the most effective for relieving OA pain [44]. However, since this study included a more comprehensive range of NSAIDs and incorporated more studies, in this research, diclofenac did not show a significant difference from other NSAIDs such as licofelone, celecoxib, lumiracoxib, tiaprofenic, and indomethacin in terms of pain relief, whether in the WOMAC pain score or the VAS pain score. It was only better than the placebo treatment group. On the contrary, etoricoxib intervention not only showed a significant reduction in pain after intervention compared to the placebo intervention but also showed a significant difference compared to a variety of non-steroidal formulations, including celecoxib and lumiracoxib. In addition, according to the SUCRA results, the best treatment for significantly reducing pain is etoricoxib. Therefore, these results support that, in terms of relieving OA pain, etoricoxib may be the preferred medication for pain relief in OA patients. This may be because etoricoxib has a higher selectivity for cyclooxygenase-2 (COX-2), so its ability to relieve pain is more outstanding [44].

In terms of improving joint function, in this study, diclofenac, celecoxib, and naproxen interventions showed better efficacy than the placebo, while other drugs did not show a significant effect better than the placebo. Therefore, when using NSAIDs to improve the joint activity function of OA patients, the above drugs are recommended first. Lin’s meta-analysis also supports this view [45].

In terms of adverse events, this study shows that adverse events after the use of NSAIDs are mostly concentrated in gastrointestinal adverse events, and some NSAIDs show cardiovascular side effects after use. Etoricoxib has an impact on increasing the incidence of cardiovascular dangerous events in patients with cardiovascular diseases, but etoricoxib and ketoprofen show better protection for the gastrointestinal tract. Curtis’s meta-analysis results show that NSAIDs can increase the risk of gastrointestinal adverse events and also increase the risk of cardiovascular adverse events [46]. This study well supplements it, providing more clinical medication options.

As a commonly used drug for the symptomatic treatment of OA, the mechanism and research on NSAIDs are also basically clear. For example, tiaprofenic can improve the inflammatory response in OA patients by reducing the levels of TNF-α, IL-1, and IL-6, thereby relieving acute or chronic pain caused by OA [47,48]. In addition, compared with indomethacin, tiaprofenic has fewer side effects and is commonly used as the first-line treatment for OA and the preferred medication for patients who cannot tolerate conventional NSAIDs [47]. Etoricoxib, as a highly selective COX-2 inhibitor, can effectively relieve the pain and discomfort of KOA patients. However, clinical pharmacological studies on etoricoxib show that although it has a lower incidence of gastrointestinal adverse events, it is more likely to lead to the occurrence of cardiovascular adverse events [47]. In terms of improving joint function, naproxen has been reported to significantly improve patients’ joint function and is not prone to serious adverse events (2.6%), and its incidence of cardiovascular adverse events is basically consistent with that of the placebo [49]. In addition, diclofenac is considered one of the most effective NSAIDs for the treatment of OA [50]. It can effectively reduce the WOAMC pain score, relieve OA pain, and restore joint function, and it is reported to be not prone to adverse safety events [46], but in this study, no significant efficacy was observed, which may be related to the insufficient sample size of the clinical report.

Adverse events caused by the treatment of NSAIDs are also an inevitable event in today’s medication use. For the evaluation of adverse events, Mozaffari A A et al. found that although ketoprofen is said to have the least toxic events in experimental animals, and the NMA results of this study also show that it has a lower incidence of gastrointestinal adverse events, all experimental animals after drug intervention were found to have gastrointestinal bleeding, erosion, and ulcers in the autopsy [8], which may be due to the inclusion of different placebo controls. In addition, trials with placebo controls for NSAIDs show that the use of NSAIDs can increase the risk of atherosclerotic thrombotic vascular events [51], such as hypertension, ischemic heart disease, and heart failure, but in this study, only etoricoxib showed a significant cardiovascular adverse reaction, which may need further clinical research to confirm. Therefore, for improving the clinical symptoms of OA, the use of etoricoxib and tiaprofenic can better improve the pain of OA; naproxen can significantly improve the joint dysfunction of OA patients, and the incidence of adverse events is low; diclofenac has a stronger effect on improving the abnormal joint activity of OA patients; ketoprofen has the least toxicity, and it can better avoid gastrointestinal events and cardiovascular adverse events.

The clinical implications of our study are significant. Clinicians can leverage our findings to make more informed decisions when prescribing NSAIDs for OA treatment. For instance, the selection of etoricoxib or ketoprofen can be guided by patients’ specific risk factors and comorbidities. This approach not only optimizes pain management but also minimizes potential adverse events. Specifically, diclofenac, despite its efficacy, should be used with caution due to its higher risk of cardiovascular events and hepatotoxicity. Additionally, naproxen has been shown to significantly improve joint function with a relatively low incidence of adverse events, making it a suitable choice for patients with functional limitations.

To ensure the robustness of our findings, we conducted a sensitivity analysis by excluding studies with a high risk of bias and re-running the network meta-analysis. The consistency of the results across these analyses indicates that our findings are reliable and can be confidently applied in clinical settings. This robustness is further supported by the alignment of our results with several clinical trials that have reported similar efficacy and safety profiles for the NSAIDs examined.

Our findings align with multiple clinical trials that have documented comparable efficacy and safety profiles for the NSAIDs examined. For example [52–54], a recent prospective multi-cohort study underscored the long-term risks associated with NSAID use, particularly among patients with comorbidities. This consistency not only reinforces the credibility of our results but also supports their application in clinical practice. Moreover, our study extends existing research by providing a more comprehensive comparison of various NSAIDs, thereby offering clinicians a broader and more nuanced basis for decision-making. Overall, the alignment of our findings with clinical trial data highlights the robustness of our conclusions and their relevance to clinical practice.

When discussing the validity of research findings, it is crucial to consider the potential impact of missing data. Missing data can introduce bias and weaken the robustness of the results. In our study, we employed suitable statistical techniques, such as multiple imputation, to address missing data and reduce its influence on the analysis. Nonetheless, the existence of missing data remains a challenge. Future research should focus on minimizing missing data through improved study design and better participant retention strategies. Furthermore, conducting sensitivity analyses can help evaluate how different assumptions about the missing data mechanism affect the robustness of the results.

While this study provides valuable insights into the efficacy and safety of NSAIDs in OA treatment, there are still several limitations to be aware of. Firstly, the outcome indicators, mainly subjective scoring ones like the VAS pain score and WOMAC scores, might not fully reflect the actual degree of pain relief and functional improvement in patients, potentially leading to bias. Future research should include more objective evaluation indicators such as imaging studies or biomarkers for a more comprehensive assessment of treatment effects. Secondly, the inconsistent dosages of NSAIDs used in different studies could affect the comparability of efficacy results. To deal with this, future research should standardize dosages across studies or carry out subgroup analyses based on dosage levels. Thirdly, some included studies had limitations including insufficient sample size and inconsistent classification methods, which may limit the generalizability of the findings. Future studies should aim for larger sample sizes and use consistent diagnostic criteria and classification methods to ensure more robust and reliable results. Moreover, the possibility of publication bias can’t be completely ruled out as some studies might have been excluded due to incomplete or unextractable data. Future research should focus on long - term studies to better understand the balance between efficacy and safety of different NSAIDs, and explore personalized treatment approaches based on patient - specific risk factors to enhance clinical outcomes.

Addressing the limitations of preclinical studies is vital for effectively translating research findings into clinical practice. Preclinical studies, such as animal experiments, can offer valuable insights into the mechanisms of action and potential efficacy of NSAIDs. However, they may not fully capture the complexity of human pathophysiology and the variability in patient responses. For instance, some NSAIDs have shown promising results in animal models, but clinical trials have revealed significant differences in efficacy and safety profiles when applied to human populations. Hence, it’s crucial to take into account both preclinical and clinical data when making treatment decisions. In the future, research should concentrate on long - term studies to gain a better understanding of the balance between efficacy and safety across different NSAIDs. This involves exploring the potential long - term impacts of NSAIDs on OA symptoms and structural changes, which have largely remained unexplored beyond short - term studies. Moreover, looking into personalized treatment approaches based on patient - specific risk factors could further enhance clinical outcomes. For example, pharmacogenetics might provide insights into how individual genetic profiles affect responses to NSAIDs, potentially enabling more tailored treatment plans. In addition, exploring the role of multimodal analgesia and incorporating patient preferences into treatment planning could improve adherence and satisfaction.

## Conclusion

This study provides a comprehensive evaluation of the efficacy and safety of various nonsteroidal anti-inflammatory drugs (NSAIDs) in treating osteoarthritis (OA). Etoricoxib, tiaprofenic acid, naproxen, and diclofenac were found to be more effective in improving clinical symptoms, while ketoprofen exhibited a lower incidence of adverse events. These findings have significant clinical implications, offering clinicians evidence-based guidance for selecting appropriate NSAIDs based on individual patient needs. Sensitivity analysis confirmed the robustness of our results, which are consistent with existing clinical trials. However, further research is needed to fully validate these findings and explore personalized treatment approaches. Future studies should focus on long-term evaluations and the development of tailored treatment strategies to enhance clinical outcomes.

## Supporting information

S1 Fig SUCRA data. (TIF)

S2 Fig VAS Score. (TIF)

S3 Fig WOMAC Score. (TIF)

S4 Fig Cardiovascular AE. (TIF)

S5 Fig Gastrointestinal AE. (TIF)

S6 Fig Any AE. (TIF)

S1 File Raw data extraction form. (XLSX)

S2 File Raw data registration form. (XLSX)

S3 File Raw data extraction. (XLSX)

S4 File Cochrane Risk of Bias Instrument data. (XLSX)

S5 File Literature search strategy. (DOCX)

S6 File PRISMA screening table. (XLSX)

S7 File Amstar II. (PDF)

S8 File PRISMA_2020_checklist. (PDF)
