# Supplementary figures and images for: Assessing the efficacy and safety of different nonsteroidal anti-inflammatory drugs in the treatment of osteoarthritis: A systematic review and network meta-analysis based on RCT trials
Source: PLoS One. 2025 May 7;20(5):e0320379. doi: 10.1371/journal.pone.0320379 (PMC12057957; doi:10.1371/journal.pone.0320379)

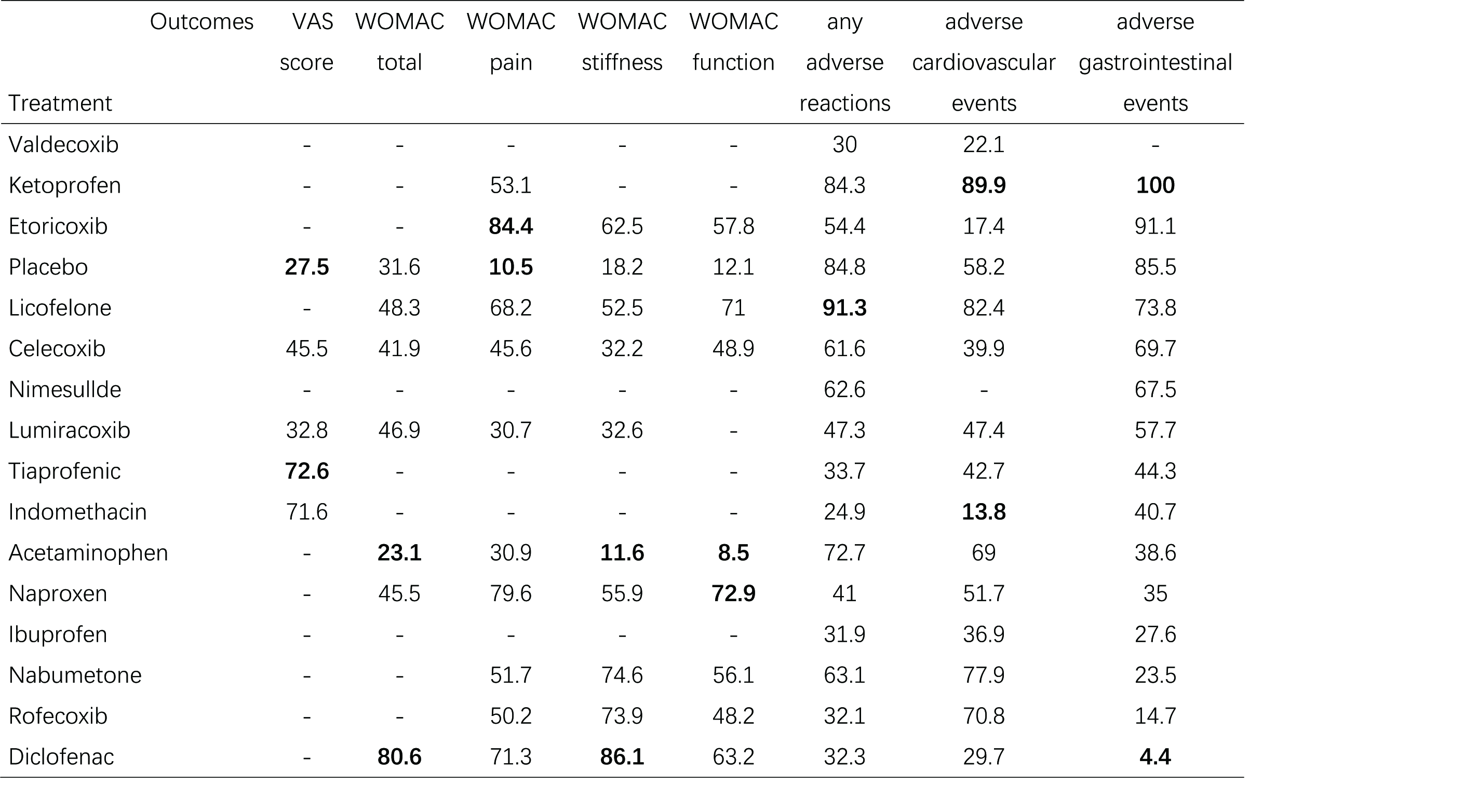

Supplement: S1 Fig — (TIF) [file pone.0320379.s001.tif]

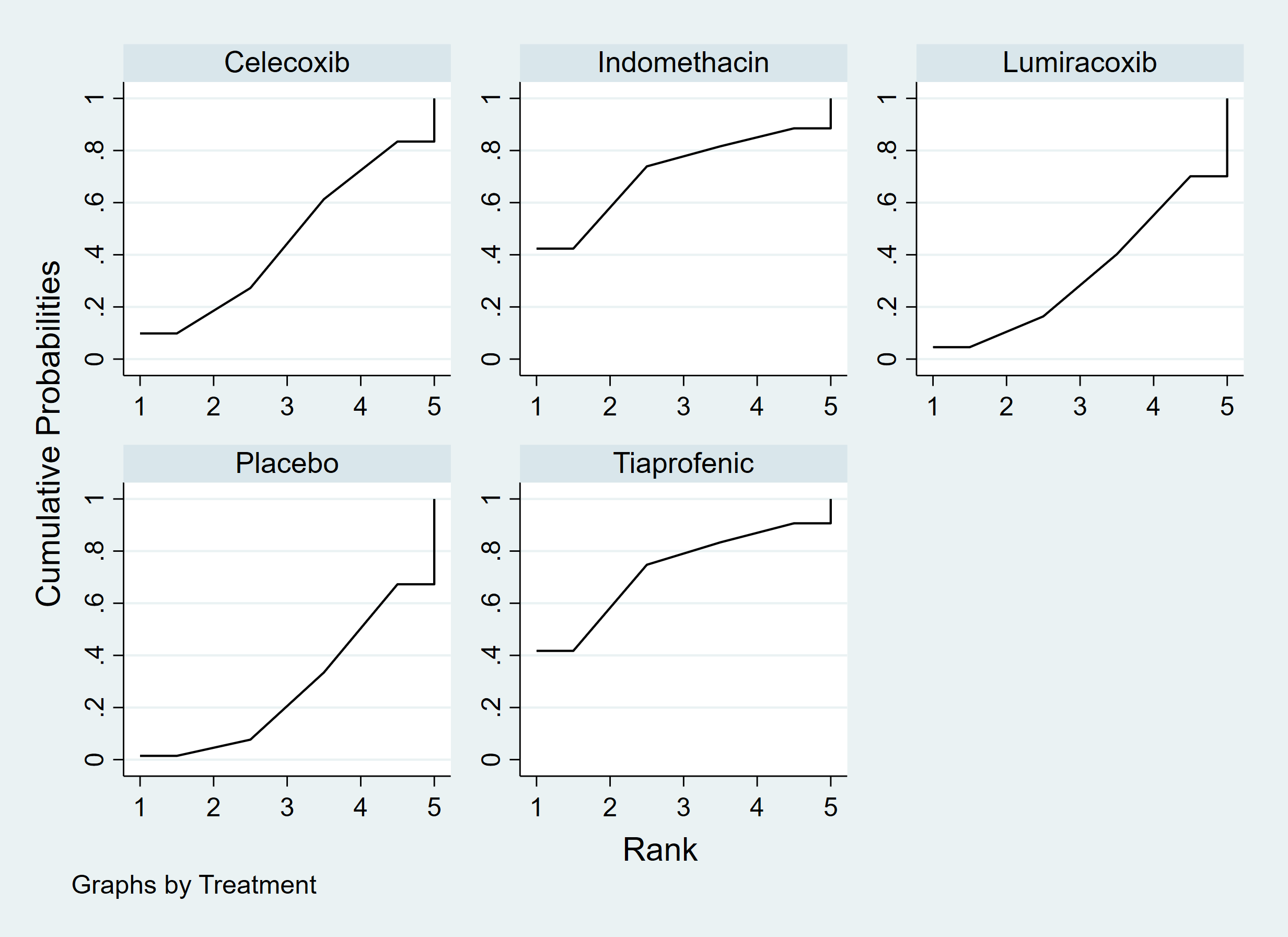

Supplement: S2 Fig — (TIF) [file pone.0320379.s002.tif]

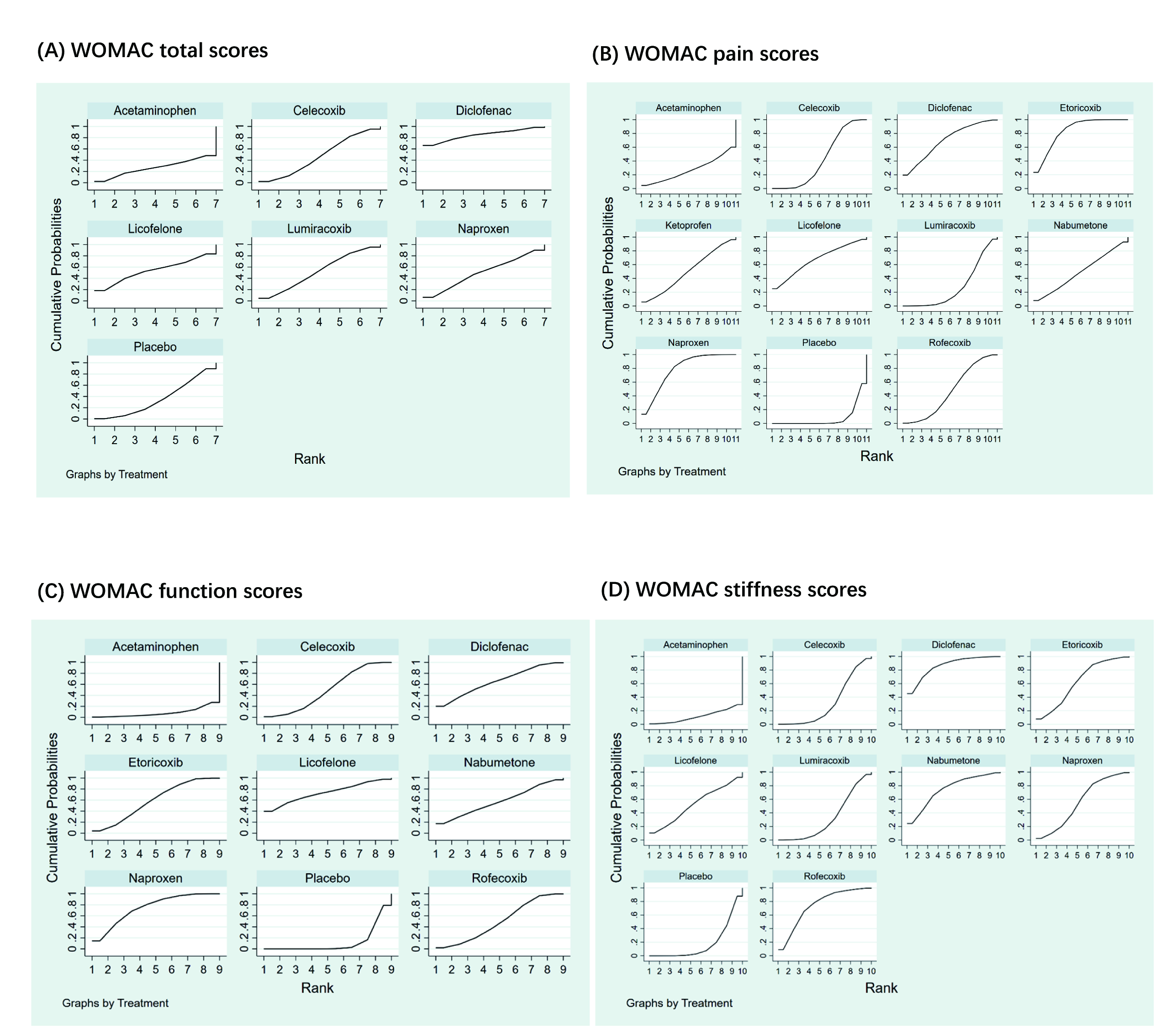

Supplement: S3 Fig — (TIF) [file pone.0320379.s003.tif]

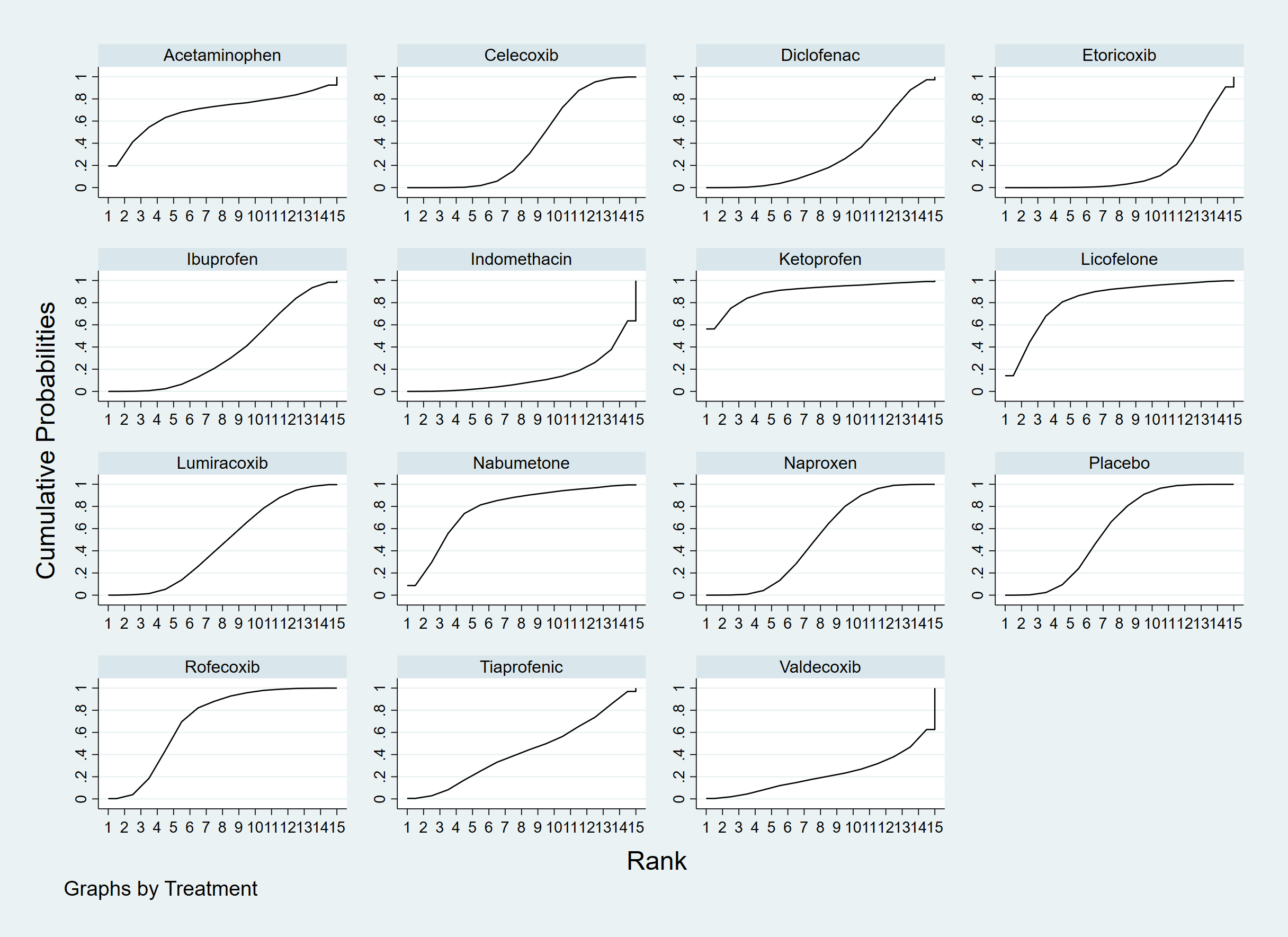

Supplement: S4 Fig — (TIF) [file pone.0320379.s004.tif]

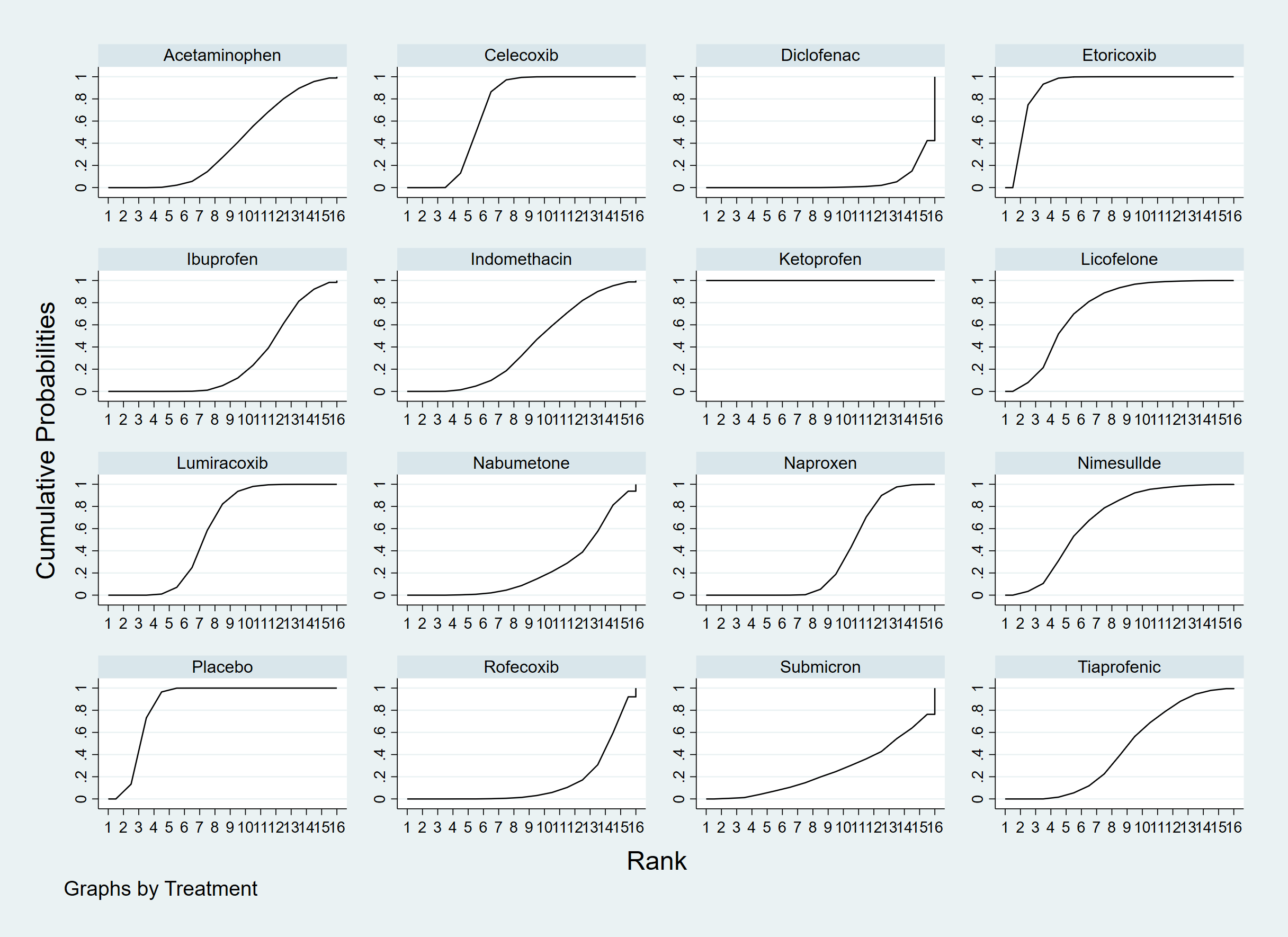

Supplement: S5 Fig — (TIF) [file pone.0320379.s005.tif]

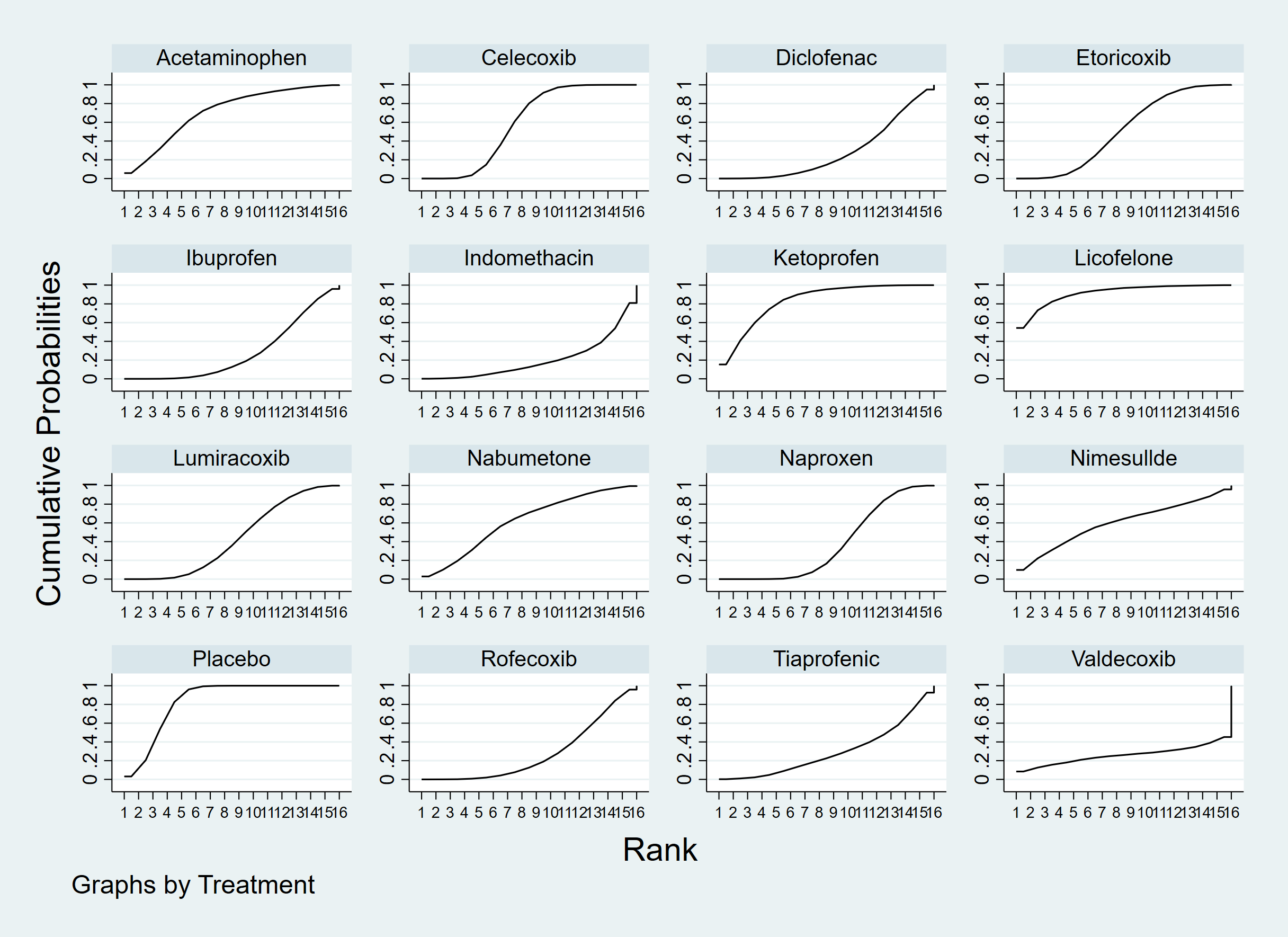

Supplement: S6 Fig — (TIF) [file pone.0320379.s006.tif]
