# Supplementary material for: Assessing the efficacy and safety of different nonsteroidal anti-inflammatory drugs in the treatment of osteoarthritis: A systematic review and network meta-analysis based on RCT trials
Source: PLoS One. 2025 May 7;20(5):e0320379. doi: 10.1371/journal.pone.0320379 (PMC12057957; doi:10.1371/journal.pone.0320379)
Supplement: S5 File — (DOCX) [file pone.0320379.s011.docx]

| **Literature Search Strategy** | |
| --- | --- |
| **Pubmed**  **1219** | ((("Osteoarthritis"[Mesh]) OR ((((((((((Osteoarthritides[Title/Abstract]) OR (Arthritis, Degenerative[Title/Abstract])) OR (Arthritides, Degenerative[Title/Abstract])) OR (Degenerative Arthritides[Title/Abstract])) OR (Degenerative Arthritis[Title/Abstract])) OR (Osteoarthrosis[Title/Abstract])) OR (Osteoarthroses[Title/Abstract])) OR (Osteoarthrosis Deformans[Title/Abstract])) OR (Arthrosis[Title/Abstract])) OR (Arthroses[Title/Abstract]))) AND ((Anti-Inflammatory Agents, Non-Steroidal[MeSH Terms]) OR (((((((((((((((((((((((((Non-Steroidal Anti-Inflammatory Agent[Title/Abstract]) OR (Agent, Non-Steroidal Anti-Inflammatory[Title/Abstract])) OR (Anti-Inflammatory Agent, Non-Steroidal[Title/Abstract])) OR (Non Steroidal Anti Inflammatory Agent[Title/Abstract])) OR (NSAID[Title/Abstract])) OR (Nonsteroidal Anti-Inflammatory Agent[Title/Abstract])) OR (Agent, Nonsteroidal Anti-Inflammatory[Title/Abstract])) OR (Anti-Inflammatory Agent, Nonsteroidal[Title/Abstract])) OR (Nonsteroidal Anti Inflammatory Agent[Title/Abstract])) OR (Anti Inflammatory Agents, Nonsteroidal[Title/Abstract])) OR (Antiinflammatory Agents, Non Steroidal[Title/Abstract])) OR (Antiinflammatory Agents, Nonsteroidal[Title/Abstract])) OR (Nonsteroidal Antiinflammatory Agents[Title/Abstract])) OR (Non-Steroidal Anti-Inflammatory Agents[Title/Abstract])) OR (Non Steroidal Anti Inflammatory Agents[Title/Abstract])) OR (Nonsteroidal Anti-Inflammatory Agents[Title/Abstract])) OR (Nonsteroidal Anti Inflammatory Agents[Title/Abstract])) OR (NSAIDs[Title/Abstract])) OR (Analgesics, Anti-Inflammatory[Title/Abstract])) OR (Anti-Inflammatory Analgesics[Title/Abstract])) OR (Aspirin-Like Agents[Title/Abstract])) OR (Aspirin Like Agents[Title/Abstract])) OR (Aspirin-Like Agent[Title/Abstract])) OR (Agent, Aspirin-Like[Title/Abstract])) OR (Aspirin Like Agent[Title/Abstract])))) AND ((randomized controlled trial[Publication Type] OR randomized[Title/Abstract] OR placebo[Title/Abstract])) |
| **Web of Science**  **2757** | 1: (((((((((((((TS=(Osteoarthritis)) OR TS=(OA)) OR TS=(Osteoarthritis, Spine)) OR TS=(Osteoarthritis, Knee)) OR TS=(Osteoarthritis, Hip)) OR TS=(KOA)) OR TS=(Hip, Osteoarthritis)) OR TS=(Knee, Osteoarthritis)) OR TS=(Spine, Osteoarthritis)) OR TS=(arthritis)) OR TS=(degenerative osteoarthrosis)) OR TS=(osteoarthritis, OA)) OR TS=(OA, osteoarthritis)) OR TS=((osteoarthritis)) and Preprint Citation Index (Exclude – Database) Results: 438612  2: ((((((((TS=(NSAIDs)) OR TS=(Nonsteroidal Antiinflammatory Drugs)) AND TS=(Non steroidal anti-inflammatory drugs)) AND TS=(nsaid)) AND TS=(nsaid)) AND TS=(nsaids)) AND TS=(nsaid s)) AND TS=(non-steroidal anti-inflammatory drugs)) AND TS=(non-steroidal anti-inflammatory drugs,NSAIDs) and Preprint Citation Index (Exclude – Database) Results: 366  3: ((((((((TS=(randomized controlled trial)) OR TS=(controlled clinical trial)) OR TS=(randomized)) OR TS=(randomised)) OR TS=(placebo)) OR TS=(sham)) OR TS=(randomly)) OR TS=(trial)) OR TS=(groups) and Preprint Citation Index (Exclude – Database) Results: 8169825  4: (((#1) AND #2) AND #3) and Preprint Citation Index (Exclude – Database) Results: 2757 |
| **Cochrane**  **1877** | #1 MeSH descriptor: [Osteoarthritis] explode all trees 10843  #2 (bone):ti,ab,kw (Word variations have been searched) 69111  #3 (skeleton):ti,ab,kw (Word variations have been searched) 950  #4 (articulation):ti,ab,kw (Word variations have been searched) 1381  #5 (joint):ti,ab,kw (Word variations have been searched) 50511  #6 (inflammation):ti,ab,kw (Word variations have been searched) 54544  #7 #2 OR #3 69515  #8 #4 OR #5 51668  #9 #6 AND #7 AND #8 523  #10 #9 OR #1 11287  #11 (NSAIDs):ti,ab,kw (Word variations have been searched) 8172  #12 (nsaid):ti,ab,kw (Word variations have been searched) 8163  #13 (non-steroidal anti-inflammatory drugs):ti,ab,kw (Word variations have been searched) 10519  #14 (Nonsteroidal Antiinflammatory Drugs):ti,ab,kw (Word variations have been searched) 13151  #15 (Non steroidal anti-inflammatory drugs):ti,ab,kw (Word variations have been searched) 10764  #16 #11 AND #12 AND #13 AND #14 AND #15 3076  #17 #10 AND #16 1877 |
| **Embase**  1815 | #24. #15 AND #22 AND #23 1,815  #23. 'randomized controlled trial'/exp 820,967  #22. #16 OR #17 OR #18 OR #19 OR #20 OR #21 1,549,197  #21. 'non-steroidal anti-inflammatory drugs' 20,467  #20. 'nsaid' 26,050  #19. 'non steroidal anti-inflammatory drugs' 20,461  #18. 'nonsteroidal antiinflammatory drugs' 4,329  #17. 'nsaids' 43,346  #16. 'nonsteroid antiinflammatory agent'/exp 1,540,698  #15. #1 OR #2 OR #3 OR #4 OR #5 OR #6 OR #7 OR #8 OR 506,496  #9 OR #10 OR #11 OR #12 OR #13 OR #14  #14. 'osteoarthritis':ab,ti 126,632  #13. 'oa osteoarthritis oa':ab,ti 4  #12. 'osteoarthritis oa':ab,ti 44,161  #11. 'degenerative osteoarthrosis':ab,ti 47  #10. 'arthritis':ab,ti 318,469  #9. 'spine osteoarthritis':ab,ti 135  #8. 'knee osteoarthritis':ab,ti 26,167  #7. 'hip osteoarthritis':ab,ti 4,762  #6. 'koa':ab,ti 3,361  #5. 'osteoarthritis hip':ab,ti 126  #4. 'osteoarthritis knee':ab,ti 939  #3. 'osteoarthritis spine':ab,ti 2  #2. 'oa':ab,ti 75,836  #1. 'osteoarthritis'/exp 172,295  ....................................................... |
