# Supplementary material for: Assessing the efficacy and safety of different nonsteroidal anti-inflammatory drugs in the treatment of osteoarthritis: A systematic review and network meta-analysis based on RCT trials
Source: PLoS One. 2025 May 7;20(5):e0320379. doi: 10.1371/journal.pone.0320379 (PMC12057957; doi:10.1371/journal.pone.0320379)
Supplement: S8 File — (PDF) [file pone.0320379.s014.pdf]

PRISMA 2020 Checklist

Section and Topic	Item #	Checklist item	Location where item is reported
**TITLE**			
Title	1	The report is identified as a network meta-analysis	1
**ABSTRACT**			
Abstract	2	The structured abstract includes objectives, study design and methods, results and conclusions.	2
**INTRODUCTION**			
Rationale	3	The report is describe the rationale for the review in the context of existing knowledge.	3
Objectives	4	The report provide an explicit statement of the review addresses.	4
**METHODS**			
Eligibility criteria	5	The report has clearly stated in the text the inclusion and exclusion criteria for the synthesis	5
Information sources	6	The report has noted all databases searched or consulted to identify research and the date each source was last searched.	6
Search strategy	7	The report has described the complete search strategy for all databases, including any filters and restrictions used.	7
Selection process	8	The methodology used to determine whether a study met the review's inclusion criteria has been clearly described in the body of the report, and two reviewers worked independently and screened each record and retrieved literature	8
Data collection process	9	The report has described the methodology used to collect the data, including the fact that two reviewers collected data from each report and worked independently.	9
Data items	10a	It determines which outcomes to collect through the inclusion exclusion criteria described above.	10a
	10b	Participants were all patients with OA, interventions were all NSAIDs, and funding sources excluded all drug company-funded articles	10b
Study risk of bias assessment	11	Two researchers working independently used the RoB2 risk of bias evaluation tool to assess the risk of bias for all included studies	11
Effect measures	12	Each result uses the mean difference as a composite or expression of the results.	12
Synthesis methods	13a	This report uses Excel software to tabulate the characteristics of the study interventions and compare them to the planned groups for each review	13a
	13b	For all missing data we excluded them according to the exclusion criteria.	13b
	13c	The results are visualised through ROC curves and rank plots.	13c
	13d	Bayesian net meta-analysis was performed using Stata 17.0. Dichotomous variables were expressed as Oddsratio (OR) as the effect analysis statistic, and continuous variables were expressed as mean difference (MD) with 95% confidence interval (CI) calculated. If the units of continuous variables were not uniform, standardised mean difference (SMD) was used to eliminate variability.	13d
	13e	If there is an open-loop structure in the network diagram, the consistency model is chosen. If there is a closed-loop structure in the network diagram, the consistency of the outcome indicators is tested by inconsistency test. The consistency model was used when P>0.05 indicated good agreement between direct and indirect evidence.	13e
	13f	The article uses a funnel plot for sensitivity analysis	13f
Reporting bias assessment	14	For missing data, we removed them before inclusion, and bias due to missing data is exactly what this study will further refine in the future	14
Certainty assessment	15	Bayesian net meta-analysis was performed using Stata 17.0. Dichotomous variables were presented as Oddsratio (OR) as the effect analysis statistic, and continuous variables were presented as mean difference (MD), and 95% confidence intervals (CI) were calculated.	15

PRISMA 2020 Checklist

Section and Topic	Item #	Checklist item	Location where item is reported
**RESULTS**			
Study selection	16a	This report describes the results of the search and screening process, using a search flowchart.	16a
	16b	Studies that appeared to meet the inclusion criteria but were excluded are not cited in this report	16b
Study characteristics	17	Each of the included studies was an RCT of OA treated with NSAIDs	17
Risk of bias in studies	18	This study assessed the risk of bias for each included study by RoB2.0.	18
Results of individual studies	19	The report provides effect estimates and their precision (confidence intervals) for each study and uses graphical presentations.	19
Results of syntheses	20a	The vast majority of projects were assessed as low risk. All 31 used randomised allocation methods. In terms of overall assessment, a total of 2 randomised controlled trials were high risk. 1 randomised controlled trial had a randomisation process that did not provide for specific measures , 1 randomised controlled trial did not refer to specific interventions for deviation from expectations , 2 randomised controlled trials had outcome data that could be at risk of missing points , and the selection of reported outcomes in 4 randomised controlled trials was were not sufficiently standardised and were therefore judged to be of unclear risk. With the exception of the above, all randomised controlled trials were assessed as low risk.	20a
	20b	The report conducts meta-analyses and lists summary estimates for each meta-analysis with their precision (confidence intervals) and measures of statistical heterogeneity.	20b
	20c	It was not possible to present all findings on the possible causes of heterogeneity between study outcomes.	20c
	20d	Sensitivity analyses are carried out in this report in the form of funnel plots and presented as results.	20d
Reporting biases	21	The report has assessed the risk of bias due to missing results (caused by reporting bias) for each synthesis assessed.	21
Certainty of evidence	22	The report has assessed the certainty (or credibility) of the evidence for each of the findings.	22
**DISCUSSION**			
Discussion	23a	The report has provided a general interpretation of the results in the light of other evidence.	23a
	23b	The report has discussed the limitations of the evidence included in the review.	23b
	23c	The report has discussed the limitations of the review process used.	23c
	23d	The report has discussed the implications of the results for practice, policy and future research.	23d
**OTHER INFORMATION**			
Registration and protocol	24a	The programme is registered in the Prospective Registry for International Systematic Evaluation (PROSPERO) (registration number CRD42024552748)	24a
	24b	Access to the review protocol can be obtained from https://www.crd.york.ac.uk/PROSPERO	24b
	24c	No changes have been made to the information provided at registration or in the agreement.	24c
Support	25	This work was supported by the State Administration of Traditional Chinese Medicine Science and Technology Department-Zhejiang Provincial Administration of Traditional Chinese Medicine Co-construction of Key Projects And Zhejiang Provincial Natural Fund Projects (No. gzy-zj-kj-23093 and Y23H270001) from Dr.Wang	25
Competing interests	26	There can be no competing interests with the reviewing authors.	26

PRISMA 2020 Checklist

Section and Topic	Item #	Checklist item	Location where item is reported
Availability of data, code and other materials	27	All data is publicly available from https://pubmed.ncbi.nlm.nih.gov/	27

From: Page MJ, McKenzie JE, Bossuyt PM, Boutron I, Hoffmann TC, Mulrow CD, et al. The PRISMA 2020 statement: an updated guideline for reporting systematic reviews. BMJ 2021;372:n71. doi: 10.1136/bmj.n71
